# Knowledge of pharmacy workers on antihypertensive and anticonvulsant drugs for managing pre-eclampsia and eclampsia in Bangladesh

**DOI:** 10.1186/s12913-020-05685-6

**Published:** 2020-09-07

**Authors:** Shongkour Roy, Kanij Sultana, Sharif Mohammed Ismail Hossain, Pooja Sripad, Charlotte E. Warren

**Affiliations:** 1 Population Council, Dhaka, Bangladesh; 2 Population Council, Washington, DC, USA

**Keywords:** Preeclampsia, Eclampsia, Pharmacy workers, Antihypertensives, Anticonvulsant, MgSO_4_, Knowledge

## Abstract

**Background:**

Pharmacy workers in Bangladesh play an important role in managing pregnancy complications by dispensing, counselling and selling drugs to pregnant women and their families. This study examined pharmacy workers’ drug knowledge and practice for pre-eclampsia and eclampsia (PE/E) management, including antihypertensives and anticonvulsants, and determine factors associated with their knowledge.

**Methods:**

A cross-sectional survey with 382 pharmacy workers in public facilities (government) and private pharmacies and drug stores assessed their knowledge of antihypertensive and anticonvulsant drugs. ‘Pharmacy workers’ include personnel who work at pharmacies, pharmacists, family welfare visitors (FWVs), sub-assistant community medical officers (SACMOs), drug storekeepers. Exploratory and multivariate logistic models were used to describe association between knowledge of medicines used in pregnancy and demographic characteristics of pharmacy workers.

**Results:**

Overall, 53% pharmacy workers interviewed were drug store owners in private pharmacies while 27% FWVs/SACMOs, who are government service providers also work as drug prescribers and/or dispensers in public facility pharmacies. Majority of pharmacy workers had poor knowledge compared to correct knowledge on both antihypertensive (77.8% vs 22.3%; *p* < 0.001) and anticonvulsant drugs (MgSO_4_) (82.2% vs 17.8%; p < 0.001). Multivariate analysis showed SACMOs and FWVs were greater than 4 times more likely to have correct knowledge on anti-hypertensives (AOR = 4.2, 95% CI:1.3–12.3, *P* < 0.01) and anticonvulsant drugs (AOR = 4.9, 95% CI:1.3–18.1, P < 0.01) compared to pharmacists. Pharmacy workers who had received training were more likely to have correct knowledge on antihypertensive and anticonvulsant drugs than those who had no training.

**Conclusions:**

Pharmacy workers’ knowledge and understanding of antihypertensive and anticonvulsant drugs, particularly for prevention and management of PE/E is limited in Bangladesh. Most pharmacies surveyed are private and staffed with unskilled workers with no formal training on drugs. Expansion of maternal and newborn health programs should consider providing additional skills training to pharmacy workers, as well as regulating these medicines at informal pharmacies to mitigate any harmful practices or adverse outcomes of unauthorized and incorrectly prescribed and used drugs. It is important that correct messaging and medicines are available as drug stores are often the first point of contact for most of the women and their families.

## Background

Hypertensive disorders in pregnancy, specifically pre-eclampsia, affect 2 to 8% of pregnancies globally and cause more than 50,000 maternal deaths each year [1]. Bangladesh has made significant gains in maternal health, of which a 71% decrease in maternal deaths from 1975 to 2014 is foremost [2]. Despite this progress, women in Bangladesh have a 1 in 240 lifetime risk of maternal death [3]. The two main causes of maternal mortality are postpartum hemorrhage and pre-eclampsia and eclampsia (PE/E), both of which are preventable [4, 5]. Pre-eclampsia causes 24% of maternal deaths in Bangladesh and is the second largest contributor to the country’s maternal mortality rate [6]. Pre-eclampsia is defined as high blood pressure and protein in the urine in pregnant women of over 20 weeks’ gestation [7]. The Bangladesh Demographic and Health Survey 2011 (BDHS 2011) reported that 32% of adult women 35 years of age and older who were tested were hypertensive, but only 21% of women over the age of 35 had been informed of their hypertension by a doctor, and among those women only 67% had taken medications to control it [8]. There is scant information on the incidence and prevalence of hypertension during pregnancy in Bangladesh, but one hospital-based study reported a prevalence of hypertension during pregnancy of 7.5% [9].

A critical health system priority for optimum care of pregnant women is ensuring enough quality drugs are available at all levels of a health system, and that individuals responsible for prescribing and dispensing these drugs have appropriate and correct knowledge. Management of PE/E involves controlling moderate and severe blood pressure with antihypertensive drugs and preventing seizures and progression to eclampsia with anticonvulsant drugs such as magnesium sulphate (MgSO_4_). Local health workers or pharmacy employees are crucial figures in maternal health care in Bangladesh, serving as the first points of contact for many pregnant women and their families who seek counseling or medication [10–13].

There are approximately 114,052 licensed retail pharmacies and drug shops [14] in Bangladesh and an approximately equivalent number of unlicensed retail drug shops selling “over-the-counter” drugs [8]. A wide range of individuals prescribe, dispense, or sell drugs over the counter for maternal health problems, often unregulated [15–17]. Registered pharmacists, diploma pharmacists (with a 3-year diploma in pharmacy) [18], pharmacy technicians, public health care providers–e.g. nurses, sub-assistant community medical officers (SACMOs) and family welfare visitors (FWVs)--along with untrained or informally trained providers and drug shop owners all prescribe or provide drugs to pregnant women in Bangladesh. In this study, ‘pharmacy workers’ are defined not only as registered pharmacists, pharmacist technicians, diploma pharmacists, and authorized health providers, including FWVs, and SACMOs, but informally trained personnel as well, including drug store and shop clerks, managers, and proprietors who provide medications from a drug store.

FWVs and SACMOs provide primary health care (PHC) services to the general populace including pregnant women. These two PHC provider cadres are trained to detect PE/E by checking blood pressure and urine protein and are authorized to administer a loading dose of MgSO_4_ (10 mg) intramuscular injection in severe cases, and then refer women to a referral hospital [19]. Drug store clerks and managers are not trained in PE/E nor hypertension detection, nor any other medical indications, and should not prescribe or administer medications, but it appears they often informally learn about medical prescription from either individuals with a medical degree, unqualified health care providers, or inherit and learn their trade informally [20]. A substantial proportion of drug store clerks and managers also appear to receive ‘dispensing knowledge’ from representatives of private pharmaceutical companies. A recent study of private drug stores and shops in Rajshahi, Bangladesh reveals that most medicines were dispensed irrationally without any prescription, and that over-the-counter dispensing of many drugs with low safety profiles is common [21]. Prescribing, selling, and dispensing drugs over-the-counter by drug store clerks is common in rural Bangladesh, and they invariably dispense and sell drugs upon patient request without any form of prescription [20].

These individuals—both trained local health workers and drug shop employees—constitute a broad informal coalition of trained and untrained individuals playing an integral role in maternal health care in Bangladesh, through their counseling as well as prescribing, selling, and dispensing of drugs to pregnant women and their families [15]. Although policy requires certification from the Pharmacy Council of Bangladesh (PCB) for local private drug stores, it is generally ignored, and these private stores are largely unregulated [13]. PCB, an autonomous organization of the Ministry of Health and Family Welfare (MoHFW), is the regulating authority for pharmacy education and practice, in addition to others [22]. PCB provides three types of pharmacy registration certifications: ‘A’-grade registration certificate (for a graduate in pharmacy from any approved university- B Pharm, ‘B’-grade registration certificate (for a three-year diploma in pharmacy from any approved Institute of Health Technology (IHT)- diploma in pharmacy, and ‘C’-grade registration certificate course (having, for 10th grade education and three-month training with successful completion of an exam-pharmacy certificate registration course. No A, B, or C registered pharmacy personnel are permitted to prescribe drugs, and are only supposed to counsel patients and dispense drugs prescribed by authorized health care providers [22, 23]. PCB also certifies pharmacists and may also approve any course, curriculum, or training materials for pharmacy workers [23].

Pregnant women with hypertension require close monitoring in addition to antihypertensive drugs according to clinical guidelines, with administration of MgSO_4_ if pre-eclampsia progresses from moderate to severe PE/E. Four antihypertensive drugs are recommended by WHO for use during pregnancy: alpha methyldopa, labetalol, nifedipine, and hydralazine [24–26]. While it is important to control blood pressure at around 150/90 mmHg or lower during pregnancy, many women not on appropriate medication end up with rapidly increased blood pressure and eclamptic seizures. While antihypertensive drugs are essential to treat severe hypertension, women with pre-eclampsia and severe symptoms must be administered MgSO_4_ to prevent or manage convulsions due to eclampsia. Calcium gluconate is a proven antidote, if MgSO_4_ toxicity occurs.

This paper presents the current knowledge and practice of the staff of both public and private, unregulated pharmacies in Bangladesh’s PHC system, and corresponding informal elements, to evaluate the prescription, selling, and dispensing of drugs for PE/E prevention and management, and which contextual factors correlate to knowledge for each specific drug. This study was part of a larger project of implementation research on under-utilized maternal health interventions and commodities in Bangladesh.

## Methods

This cross-sectional survey used a structured and validated questionnaire among pharmacy workers from March 2016 to May 2016 in 12 sub-districts of four Bangladesh districts.

### Study site

Bangladesh is a low- or middle-income country (LMIC) with 64 administrative districts. This study was conducted in the four districts of Bhola, Comilla, Rangpur, and Tangail. Twelve sub-districts were selected as Ending Eclampsia project implementation sites, to typify and represent rural Bangladesh. The project conducted a landscape analysis prior to developing project interventions to strengthen selected government health facilities for PE/E detection, prevention, and management [19]. The 12 implementation sub-districts featured 116 Union Health and Family Welfare Centers (UH&FWCs), 11 Upazilla Health Complexes (UHCs), five Mother and Child Welfare Centers (MCWCs), four district hospitals, 136 government pharmacies, and more than 1500 private pharmacies or drug stores in the areas selected for the study [14].

### Study population and settings

The study population comprised pharmacy staff including pharmacists, pharmacy technicians, FWVs, and SACMOs working in both public (i.e. government) facility pharmacies and private pharmacies or drug stores selected from an allopathic pharmacy and drug store list provided by the Directorate General of Drug Administration of MoHFW of Bangladesh. Convenience sampling selected one participant each from one pharmacy or drug store within a two kilometers range of a study health care facility.

### Sample size calculation

The sample size (*n* = 404) was estimated using standard error of proportions formula with a 95% confidence interval (Z value = 1.96) using a level of precision 5%. Since there were no studies conducted for pharmacy workers’ knowledge of antihypertensive and anticonvulsant drugs in Bangladesh, we assumed 50% of pharmacy workers had knowledge on antihypertensive and anticonvulsant drugs (*p* = 0.50) and added 5% to the sample to address the non-response rate. In total, 404 pharmacy workers were approached for the survey; 384 responded affirmatively, resulting in a response rate of 95%. Among the 384 participants, two questionnaires were not completed, so 382 pharmacy workers contributed to the final analysis.

### Survey instrument development

The survey investigated pharmacy staff knowledge of antihypertensive and anticonvulsant drugs for treating PE/E. Researchers administered a structured, quantitative questionnaire through individual interviews with pharmacy workers. The structured questionnaire (available as Additional file 1) was adopted and developed through an extensive review of the literature [27–32], and included knowledge, practice and management of drug stocks of antihypertensive and anticonvulsant drugs. The questionnaire has 65 items or questions in eight sections including general participant information, knowledge of antihypertensive drugs, drug procurement and ordering, current stock, practice and management of stock, reporting, supervision, training, and tools employed in the drug store.

Instrument development, including face and content validity, was assessed by experts from the Population Council’s Institutional Review Board (IRB) and the Bangladesh Medical Research Council ethical review committee, in addition to three researchers, a pharmacist, and three clinicians including a professor of obstetrics. Consistency, clarity, understanding, and time of completion of the interview were assessed in pre-testing among 15 purposely selected pharmacy workers outside the sampling area. The initial questionnaire was developed in English and was translated to Bengali for use in the field.

### Data collection

The data collection team was comprised of trained interviewers with at least bachelor’s degree. All study investigators and enumerators successfully completed a four-day training on the objectives of the study, the specific tools and methodologies, and research ethics principles. Data collectors explained the study to each potential respondent and obtained their voluntary informed consent prior to starting each interview. Response verification was conducted by the study team, including assessing the completion of each questionnaire, logical checking, and missing data before data processing and statistical analyses.

### Variables

#### Outcome binomial variables: antihypertensive and anticonvulsant drug knowledge

A set of questions reflecting pharmacy workers’ awareness of hypertensive drugs for pregnancy developed an approximate measure of antihypertensive drug knowledge, such as, “Are you aware with the medicines used to treat hypertension?”, “Do you know what antihypertensive drugs are safe to use during pregnancy?” and “Are you aware of the medicine (Alpha Methyldopa) used for the treatment of hypertension?” A three-point index (composite score range: 0–3) assigned values to dichotomized responses for awareness, where aware (“yes”) was assigned a value of 1, and not aware (“no”), 0. The distribution of individuals divided knowledge into two categories: 0 = poor knowledge (score = 0–2), and 1 = correct knowledge (score = 3). Similarly, we constructed a summary anticonvulsant drug knowledge two-point index (score range: 0–2) of the binary responses 0 (“score 0-1: poor knowledge”) and 1 (“score 2:correct knowledge”) to two questions, “Are you aware of the medicines (e.g. magnesium sulphate) for the treatment of convulsions during pregnancy?” and “Have you heard about a drug called calcium gluconate?”

#### Explanatory variables

Explanatory variables associated with knowledge of the drugs include demographics (e.g. sex, age, pharmacy worker cadre, type of medical drug store, geographic district, and years of work experience) and correctly prescribed medicines during pregnancy. Correctly prescribed medicines during pregnancy included correct drug names stated for use in hypertension during pregnancy. We constructed a variable for correct mention of any drug used for hypertension during pregnancy with the response 1 for “yes,” if respondents mentioned any one of the four specific drugs to control hypertension in pregnancy (alpha methyldopa, labetalol, nifedipine, and hydralazine) and 0 for “no” if s/he was unaware of these drugs or gave a wrong answer. Similarly, we also created a variable if for correct mention of any safe drug for hypertension during severe PE/E, with the response 1 for “yes,” if respondents mentioned any one of the four drugs alpha methyldopa, labetalol, nifedipine, and hydralazine and 0 for “no” if s/he was unaware of these drugs or gave a wrong answer. A variable was generated for any basic medical training from the government or a private organization, including trainings for pharmacists, pharmacy technicians, medical technologists, laboratory technicians, FWVs, and medical assistants.

### Statistical analysis

Categorical variables were summarized by frequencies and percentages. Differences in the distribution of demographic characteristics, antihypertensive drugs for hypertension mentioned by pharmacy workers, and antihypertensive and anticonvulsant drug knowledge were detected by Chi-squared test or Fisher’s exact test, as appropriate. For multivariate analysis, we used multivariate models of multivariate logistic regression that included demographic factors and correctly mentioned alpha methyldopa, labetalol, nifedipine, and hydralazine for hypertension during pregnancy (the same used for univariate analysis).

A multivariate logistic model assessed the association between binomial dependent factors of pharmacy workers’ antihypertensive and anticonvulsant drug knowledge, demographic characteristics, and correct names of safe medications for treating hypertension and pre-eclampsia. We assessed the adjusted odds ratios (AORs) with different models: First, we added demographic factors (sex, age, pharmacy worker cadre, type of medical drug store, geographic district, and years of work) and training in hypertension, and finally correct names of safe medicines for hypertension in pregnancy. Stata software version 12.0 (Stata Corporation, College Station, TX, USA) was used for statistical analyses, all reported *p*-values were two tailed, and a p-value of < 0.05 was considered to be statistically significant.

### Ethical considerations

This study received ethical clearance from the Population Council’s IRB in New York (IRB protocol 693) and local ethical approval from Bangladesh Medical Research Council (BMRC, reference number BMRCINREC/2013–2016/1270). All interviewees provided full, informed, voluntary, written consent before commencing each interview. Interviewees were informed that their participation was completely voluntary, and could cease at any time, and that their identities will remain anonymous.

## Results

The background characteristics of the 382 respondents are summarized in Table 1. About one third of the study participants were 50 years of age or older, within a range of 15 to 85, and the majority were male. In private pharmacies, more than half of pharmacy staff were proprietors or owners, and about 10% were clerks or managers. In public facility (i.e government) pharmacies, about 27% were FWVs or SACMOs, and 3% were drug storekeepers; few (7%) were actual trained pharmacists. While few trained pharmacists were encountered, FWVs and SACMOs at public facilities also worked as drug dispensers in addition to their regular service provision duties. Nearly three quarters of pharmacy workers had more than 5 years’ experience (Table 1).

**Table 1:** Background characteristics of pharmacy workers

Variables	N	%
**District/ Geographic distribution**		
Comilla	124	32.5
Rangpur	80	20.9
Tangail	90	23.6
Bhola	88	23.0
**Sex**		
Male	316	82.7
Female	66	17.3
**Age (years)**		
< 30	69	18.1
30–39	114	29.8
40–49	88	23.0
> =50	111	29.1
**Number of years’ experience**		
< 1 year	15	3.9
1–5 years	87	22.8
> 5	280	73.3
**Received any basic medical training**		
No	92	24.1
Yes	290	75.9
**Type of medical drug store**		
**Public**		
District Hospital/MCWC	8	2.1
Upazila Health Complex (UHC)	21	5.5
Union Health and Family Welfare Center (UH&FWC)	114	29.8
Private		
Private pharmacies/drug stores	239	62.6
**Title of pharmacy worker**		
*Public*		
Pharmacist	28	7.3
Service providers (FWV/SACMO) additional work in pharmacy	103	27.0
Drug store keeper	12	3.1
*Private*		
Drug seller/store manager	37	9.7
Drug store owner	202	52.9
**N**	**382**	**100.0**

Table 2 shows the pharmacy workers’ basic pharmacy training. Those identified as pharmacists all had training as a pharmacist or diploma pharmacist. Service providers such as FWVs or SACMOs all had learned to dispense medications from experience on the job. Similarly, all drug storekeepers in public pharmacies, 75% of private store clerks and managers, and 27% of private store owners all learned from on the job experience. Only about 18% of private store clerks and managers and 30% of store owners reported completion of any pharmacy certificate registration course. Over half (58%) of drug store owners reported learning from informal health practitioners such as village doctors or *pollichikitshok* (Table 2).

**Table 2:** Basic training on pharmacy by type of pharmacy workers

	Public			Private	
	Pharmacist (*n* = 28) %	Service provider (FWV/SACMO) (*n* = 103) %	Drug store keeper (*n* = 12) %	Drug seller/ store manager (*n* = 37) %	Drug store owner (*n* = 202) %
**Had basic training on Pharmacy**	**24 (85.7)**	**54 (52.4)**	**6 (50.0)**	**28 (75.7)**	**178 (88.1)**
**Types of training**^a^					
Pharmacist/diploma pharmacist	100.0	0.0	0.0	0.0	0.0
Certificate course	0.0	0.0	0.0	17.9	30.3
Learned from experience	0.0	100.0	100.0	75.0	26.9
Informal healthcare (village doctor/Pollichikitshok)	0.0	0.0	0.0	7.1	58.4

^a^Multiple response

Most pharmacy workers (*n* = 382) revealed poor knowledge of both antihypertensive (77.8% vs 22.3%; *p* < 0.001) and anticonvulsant (82.2% vs 17.8%; *p* < 0.001) drugs, both worker designation or cadre (p < 0.001 vs p = 0.09) and geographic distribution or district (*p* = 0.01 vs p < 0.001) (Table 3). There were no significant differences between those who had poor versus correct knowledge of antihypertensive drugs by sex, age, and years of experience, exposure to basic medical training; however, differences were observed in correct knowledge in anticonvulsant drugs by sex and basic medical training (*p* = 0.09, and *p* = 0.003). Males and those with basic medical training had better anticonvulsant drug knowledge than females and those with no basic medical training. While both public and private pharmacy workers had poor knowledge of antihypertensive and anticonvulsant drugs, private pharmacy staff had poorer knowledge than public pharmacy staff for antihypertensive (85.8% vs 64.3%) and anticonvulsant (86.2% vs 75.5%) drugs. Among public pharmacy staff, formally trained SACMOs and FWVs had the highest levels of correct knowledge for both antihypertensive (39.8%%) and anticonvulsant (25.2%) drugs, followed by pharmacists (28.6% vs 25.0%) (Table 3).

**Table 3:** Pharmacy workers’ knowledge on antihypertensive and anticonvulsant drug by demographic and employment characteristics

Variables	Knowledge on antihypertensive					Knowledge on anticonvulsant				
	Poor (*n* = 297)		Correct (*n* = 85)		P-value	Poor (*n* = 314)		Correct (*n* = 68)		P-value
	n	%	n	%		n	%	n	%	
**Sex**					0.919^a^					0.09^a^
Male	246	77.8	70	22.2		255	80.7	61	19.3	
Female	51	77.3	15	22.7		59	89.4	7	10.6	
**Age (year)**					0.396^a^					0.38^a^
< 30	56	81.2	13	18.8		59	85.5	10	14.5	
30–39	92	80.7	22	19.3		96	84.2	18	15.8	
40–49	63	71.6	25	28.4		67	76.1	21	23.9	
> =50	86	77.5	25	22.5		92	82.9	19	17.1	
**Type of medical drug store**					< 0.001^b^					0.07^b^
*Public (n = 143)*										
District Hospital /MCWC	4	50.0	4	50.0		6	75.0	2	25.0	
UHC	16	76.2	5	23.8		16	76.2	5	23.8	
UH&FWC	72	63.2	42	36.8		86	75.4	28	24.6	
*Private (n = 239)*										
Private pharmacies/ drug stores	205	85.8	34	14.2		206	86.2	33	13.8	
**District/ geographical distribution**					0.01^a^					< 0.001^a^
Comilla	98	79.0	26	21.0		105	84.7	19	15.3	
Rangpur	51	63.8	29	36.2		67	83.8	13	16.3	
Tangail	75	83.3	15	16.7		62	68.9	28	31.1	
Bhola	73	82.9	15	17.1		80	90.9	8	9.1	
**Title of pharmacy worker**					< 0.001^b^					0.09^b^
*Public*										
Pharmacist	20	71.4	8	28.6		21	75.0	7	25.0	
Service providers (FWV/SACMO) additionally, work in pharmacy	62	60.2	41	39.8		77	74.8	26	25.2	
Drug store keeper	10	83.3	2	16.7		10	83.3	2	16.7	
*Private*										
Drug seller/ store manager	34	91.9	3	8.1		33	89.2	4	10.8	
Drug store owner	171	84.7	32	15.3		173	85.6	29	14.4	
**Years’ experience**					0.59^b^					0.36^b^
Less than 1 year	11	73.3	4	26.7		12	80.0	3	20.0	
1–5 years	71	81.6	16	18.4		76	87.4	11	12.6	
More than 5	215	76.8	65	23.2		226	80.7	54	19.3	
**Received any basic medical training**					0.32^a^					0.003^a^
No	75	81.5	17	18.5		85	92.4	7	7.6	
Yes	222	76.5	68	23.5		229	78.9	61	21.1	

Note: a: Chi-square test and b: Fisher exact test: *FWV* Family Welfare Visitor, *SACMO* Sub Assistant Community Medical Officer, *MCWC* Maternal and Child Welfare Center, *UHC* Upazila Health Complex, *UH&FWC* Union Health and Family Welfare Center

In univariate analysis, the sex of pharmacy workers was not significantly associated with knowledge of antihypertensive drugs. After multivariate adjustment, Table 4 indicates that the sex of pharmacy workers was significantly correlated with knowledge of both antihypertensive and anticonvulsant drugs. Male workers were more than four times more likely to evince correct knowledge of antihypertensive drugs (Adjusted Odds Ratio (AOR):4.2, 95% Confidence Interval (CI): 1.7–10.6, *P* < 0.05) and more than six times as likely to have correct knowledge of anticonvulsant drugs (AOR: 6.5, 95% CI: 2.1–19.9, P < 0.01) than female pharmacy workers. SACMOs and FWVs were more than four times as likely to have correct knowledge of anti-hypertensives (AOR: 4.2, 95%CI: 1.3–12.3, *P* < 0.01) and around five times more likely to have correct knowledge of the anticonvulsant drug MgSO_4_ (AOR:4.9, 95%CI: 1.3–18.1, *P* < 0.01) than pharmacists. Pharmacy workers who had received training were more likely to demonstrate correct antihypertensive and anticonvulsant knowledge than those who had not received training (AOR: 2.6, 95% CI: 1.2–5.7, *P* < 0.01) and (AOR: 3.2, 95% CI: 1.1–8.1, *P* < 0.05). Other independent variables—e.g. age, years of experience, and district—were not significantly correlated with correct knowledge of antihypertensive and anticonvulsant drugs, except Rangpur district, for antihypertensive drugs (AOR: 2.9, 95% CI: 1.4–6.2, *P* < 0.01), and Tangail district, for the anticonvulsant drug (AOR: 3.6, 95% CI: 1.7–7.6, *P* < 0.01).

**Table 4:** Odd Ratios of pharmacy workers correct knowledge of antihypertensive and anticonvulsant drugs in pregnancy by demographic and employment characteristics

Variables	Anti-hypertensive Knowledge,		Anticonvulsant Knowledge,	
	OR (95%CI)	AOR (95%CI)	OR (95%CI)	AOR (95%CI)
**Sex**				
Female	1	1	1	1
Male	0.9 (0.5–1.8)	4.2* (1.7–10.6)	2.0 (0.9–4.6)	6.5** (2.1–19.9)
**Age (year)**				
< 30	1	1	1	1
30–39	1.1 (0.5–2.2)	0.9 (0.4–2.1)	1.1 (0.5–2.6)	1.2 (0.5–3.3)
40–49	1.7 (0.8–3.7)	1.1 (0.4–2.8)	1.2 (0.5–2.8)	1.9 (0.6–5.6)
> =50	1.3 (0.6–2.7)	0.5 (0.2–1.2)	1.2 (0.5–2.8)	0.7 (0.2–2.2)
**District/ geographical distribution**				
Comilla	1	1	1	1
Rangpur	2.1* (1.1–4.0)	2.9** (1.4–6.2)	1.1 (0.5–2.3)	1.1 (0.4–2.7)
Tangail	0.7 (0.4–1.5)	0.8 (0.4–1.8)	2.5** (1.3–4.8)	3.6** (1.7–7.6)
Bhola	0.8 (0.1–1.6)	1.1 (0.5–2.5)	0.6 (0.2–1.3)	0.9 (0.3–2.4)
**Title of pharmacy worker**				
Public				
Pharmacist	1	1	1	1
Service providers (FWV/SACMO) additionally, work in pharmacy	1.6 (0.7–4.1)	4.2** (1.3–12.3)	1.0 (0.4–2.7)	4.9** (1.3–18.1)
Drug store keeper	0.5 (0.1–2.8)	0.6 (0.1–3.9)	0.6 (0.1–3.4)	0.9 (0.2–5.4)
Private				
Drug seller/store manager	0.2* (0.1–0.9)	0.1** (0.02–0.6)	0.4 (0.1–1.4)	0.5 (0.1–2.5)
Drug store owner	0.5* (0.2–1.1)	0.3* (0.1–0.9)	0.5 (0.2–1.3)	0.5 (0.2–1.6)
**Years’ experience**				
Less than 1 year	1	1	1	1
1–5 years	0.6 (0.1–2.2)	0.9 (0.2–3.8)	0.6 (0.1–2.4)	0.5 (0.1–2.2)
More than 5	0.8 (0.3–2.7)	1.2 (0.3–4.5)	0.9 (0.3–3.5)	0.7 (0.2–3.1)
**Received any basic medical training**				
No	1	1		1
Yes	1.4 (0.7–2.4)	2.6* (1.2–5.7)	3.0* (1.4–7.4)	3.2* (1.1–8.1)

Inference: * p < 0.05; ** P < 0.01; *** *P* < 0.001

Table 5 shows correlates of correct knowledge of any antihypertensive and anticonvulsant drugs use during pregnancy. Pharmacy workers evincing correct knowledge of antihypertensive drugs were 17.9 times more likely to correctly demonstrate hypertensive drug use during pregnancy than pharmacy workers with poor knowledge of antihypertensive drug (AOR: 17.9, 95%CI: 7.7–41.6, P < 0.001). Pharmacy workers with correct knowledge of anticonvulsants were 3.2 times more likely to correctly recommend anticonvulsants for convulsions during pregnancy than pharmacy workers with poor knowledge of anticonvulsant drugs (AOR: 3.2, 95%CI: 1.6–6.2, *P* < 0.001). Correct knowledge of antihypertensive (AOR: 14.6, 95%CI: 5.5–38.8, P < 0.001) and anticonvulsant (AOR: 3.0, 95%CI: 1.1–7.7, *P* < 0.01) drugs was significantly correlated with correct knowledge of any of the four antihypertensive drugs (alpha methyldopa, labetalol, nifedipine, and hydralazine) for PE/E.

**Table 5:** Correctly know any drugs such as Alpha Methyldopa, Labetalol, Nifedipine and Hydralazine for hypertension during pregnancy and MgSO_4_ for PE/E associated with correct antihypertensive and anticonvulsant knowledge using multivariate logistic model

Indicators	Antihypertensive knowledge		Anticonvulsant knowledge	
	OR (95%CI)	AOR^**〹**^ (95%CI)	OR (95%CI)	AOR^**〹**^ (95%CI)
Correctly know any drug for hypertension during pregnancy	16.5*** (7.4–36.9)	17.9*** (7.7–41.6)	3.1*** (1.5–6.2)	3.2*** (1.6–6.2)
Correctly know any drug for hypertension during SPE/E	11.4*** (4.5–28.5)	14.6*** (5.5–38.8)	2.9** (1.4–7.8)	3.0** (1.1–7.7)
Correctly know injection MgSO_4_ for management of convulsion	1.9 (0.7–5.7)	1.6 (0.6–4.8)	5.5*** (1.9–15.8)	5.6*** (2.0–15.9)
Wald chi-square (p-value) [R-square]	101.5 (p < 0.001) [48.1%]	97.3 (p < 0.001) [49.4%]	73.7 (p < 0.001) [27.8%]	74.1 (p < 0.001) [28.0%]

Inference: * p < 0.05; ** P < 0.01; *** P < 0.001; 〹 adjusted for age and districts.

## Discussion

This study investigated knowledge and practice related to antihypertensive (alpha methyldopa, labetalol, nifedipine and hydralazine) and anticonvulsant (MgSO_4_ and its antidote, calcium gluconate) drugs among pharmacy workers in selected areas of Bangladesh, particularly at the district and lower level public (i.e. government) facilities as well as private pharmacies and drug stores. It reveals complexities for the prescription of antihypertensive and anticonvulsant drugs and their dispensing staff, particularly due to the lack of availability of authorized, qualified pharmacists in public PHC facilities (e.g. government-run pharmacies). Clinical service providers are often compelled to assume pharmacist duties when registered pharmacists might not be available; they have demonstrated competencies in this capacity. Staff of private drug stores are also prescribing and dispensing the same drugs, completely unregulated, without sufficient knowledge nor any counseling capabilities [27]. To safely and appropriately dispense drugs for pregnancy complications and prevent adverse outcomes, pharmacy workers, particularly those in private pharmacies, must have specific knowledge and skills, including contraindications for medicines, and their use and risks [33, 34]. If pharmacy staffs are to effectively dispense antihypertensive or anticonvulsant drugs for management of hypertensive disorders during pregnancy, they must know which drugs are safe and effective, and what to do in case of adverse events [35–39, 41–49]. Unfortunately, information supporting antihypertensive drug use during pregnancy, including benefits and risks, is frequently lacking and contradictory among pharmacy staff in Bangladesh.

Although there is a wide range of training, skills, and experience among pharmacy workers surveyed in this study, particularly among public system health care providers, more than three-fourths of pharmacy staff interviewed demonstrated poor knowledge of antihypertensive and anticonvulsant drugs, primarily due to the number of untrained providers in private pharmacies. Except for accredited medical service providers, pharmacy staff should not diagnose nor prescribe any drugs to patients, but they do [21]. Results indicate that clients who seek medications for hypertension and convulsions during pregnancy from private pharmacies can receive limited or inappropriate counseling, and receive contraindicated drugs. Our findings are similar to other studies in retail drug shops in Bangladesh [13, 39]. A previous study reported that informal allopathic providers commonly prescribe sedatives or sleeping pills, antidepressants, and beta blockers to patients with hypertension in both rural and urban areas [27]. Another study reported that pharmacy workers not only prescribed hypertension medications [40] but menstrual regulation medications as well, and that significant numbers of private pharmacy staff had deficient knowledge in routes of administration, recommend doses, and regimens of mifepristone–misoprostol [41]. Patients seek medications for PE/E from private pharmacies instead of public facility (i.e. government) pharmacies often due to periodic stock outs at government pharmacies. This study found that only 3% of public pharmacies had a loading dose of MgSO_4_ injection for PE/E management [19].

While most pharmacy staff surveyed in this study’s four districts work in private pharmacies or drug stores, there were significant disparities between public and private pharmacy workers’ knowledge of antihypertensive drugs. Only one-fourth of the pharmacy staff in this study demonstrated correct knowledge for dispensing drugs such as alpha methyldopa, labetalol, nifedipine, and hydralazine that are safe for controlling pregnant women’s high blood pressure during severe PE/E, in addition to providing correct prescribing information for these drugs. Among those surveyed who demonstrated correct knowledge of antihypertensive drugs, only one in 10 correctly dispense MgSO_4_ injection for women with severe PE/E, usually in public facilities. Those correctly dispensing (as well as prescribing) antihypertensives are primarily service providers (FWVs or SACMOs) who are also practicing pharmacists. These service providers’ superior knowledge may be due to their training on eclampsia and hypertension from the Ending Eclampsia project. Some public pharmacy staff (mostly service providers) prescribe and dispense MgSO_4_- a safe drug for convulsions, while others prescribe diazepam, and phenobarbitone, two drugs not recommended by WHO and not approved for this use in Bangladesh [26].

This study reveals that private pharmacies and drug shops are mostly unregulated, without any formal accreditation. This demands urgent attention. In LMICs such as Bangladesh, pharmacies are often a primary means of access to health care, but pharmacy workers frequently lack essential information and necessary resources to fulfill complex challenges and needs at the nexus of patient care and safe and effective use of medications.

Public sector pharmacy workers demonstrate greater knowledge of antihypertensive medications likely because of their formal training and accreditation processes that private pharmacy and drug store staff are not provided [39]. This study’s multivariate model found that FWVs and SACMOs with correct knowledge of anticonvulsant drugs were also more likely to correctly prescribe MgSO_4_ injection for convulsions. Except for FWVs and SACMOs who also work at public pharmacies, pharmacy workers do not prescribe MgSO_4_ for convulsions but instead dispense the drug, but these pharmacy workers are prescribing as well as dispensing antihypertensive drugs, despite their lack of authorization. Similar findings were reported by John Parr et al. in 2012 in a study of informal allopathic doctors in Bangladesh [27].

A larger challenge for volunteer and community health workers, along with pharmacy workers, is their limited knowledge of PHC beyond drug distribution [42]. Although officially prohibited, many drug sellers provide prescription medications in addition to diagnostic and therapeutic medical advice. One study in Bangladesh found informal practitioners as immediate, essential health care providers among the poor, but with a majority lacking essential training, knowledge, or other capacities to provide basic curative services, and thus contributing to contraindicative drug use [43]. SACMOs and FWVs are associated in this study with significantly better knowledge of antihypertensive and anticonvulsant drugs than all other health care cadres, even pharmacists, likely due to their broad, basic training of 3 years and 18 months, respectively, and clinical practice covering obstetric complications. General pharmacy staff—store clerks, managers, and proprietors—receives no form of medical training and receive no kind of accreditation. Given the unregulated dispensing of drugs in Bangladesh, including prescriptions, it is imperative to reconsider the country’s pharmacy accreditation process and include private proprietors in official drug policy and a comprehensive regulatory process.

While pharmacy staff should not prescribe any drug, it practices it is a common occurrence, and we recommend significant, effective measures to regulate private pharmacies’ dispensation of medications much more closely. Not merely developing pharmacy workers’ health care knowledge beyond medicine dispensing, but integrating them within the formal system as PHC providers with specified responsibilities and formal support should be considered in future policy and programming [44]. Officials in Bangladesh should also implement regulatory policy with routine processes to strengthen the capacities of private pharmacy staffs, particularly their knowledge of prescription medication indications. The public health system should strongly consider adopting, and adapting, innovative education programs already implemented in the country. One private organization, The Vennue Foundation, is implementing a pharmacy workforce education program that equips local staff with the knowledge and skills for effective provision of quality, patient-centered pharmaceutical care [45].

Generally, pharmacy workers in Bangladesh are male, reflected in this study sample as well as others [27]. The female pharmacy workers in this sample are primarily service providers (e.g. FWVs) with other public health responsibilities. Bangladesh’s private and public pharmacy staffing gender disparity differs markedly from the greater South East Asia region, where the majority—54%--of staff is female in independently operated community pharmacies [46]. Bangladesh’s rural female community health workforce, with the exception of family planning programs, is small due to cultural and familial restrictions on women’s employment outside the home [47]. One study has shown that cultural and religious beliefs also create barriers to women’s care during pregnancy, especially when services are only available from male health care workers [48]. To establish an equitable workforce that can provide the most efficacious services for women, who prefer health care services from women, pharmacy owners should endeavor to create more opportunities for female employees. The higher proportion of male pharmacy staff demonstrating correct knowledge of antihypertensive and anticonvulsant drugs than female staff in this study is, at least in part, likely an effect of the proportionally smaller sample of female pharmacists (FWVs with additional responsibilities), exacerbated by women’s limited opportunities for education, training, and equitable employment as pharmacy workers [49, 50]. Although the proportion of female pharmacy workers in South East Asia has increased over the last few decades [51], such a scenario is unlikely in Bangladesh until greater political will is manifested.

Although course curricula for pharmacist diplomas and certificates include various drug categories, practical training for general pharmacy staff including antihypertensive and anticonvulsant drugs for PE/E management, along with other drugs used by pregnant and postpartum women, needs to be developed, officially adopted, implemented, and sustained. The results of this study demonstrate the need for training and continuing education for all pharmacy staff. Job aids and simple instructions, for ready reference by pharmacy staff at all levels, throughout Bangladesh, would also be both useful and beneficial. If Bangladesh’s regulatory authorities incorporate practical information on antihypertensive and anticonvulsant drugs in current and future training modules for all cadres of pharmacy staff, with universal monitoring and support to all pharmacy workers, it can result in improved use and maternal health outcomes for antihypertensive and anticonvulsant drugs.

### Limitation

This study should be considered with some limitations, particularly its comparability with other studies and the ability to make causal inferences due to a cross-sectional design. Similar studies, particularly of pharmacy workers’ knowledge of antihypertensive and anticonvulsant drugs, are not available, so we were unable to directly compare findings. Future research should consider longitudinal designs and explore the roles of pharmacy workers in other contexts, such as poor urban settings, where density and patronage of private drug sellers, as initial points of health care, are high.

## Conclusions

Pharmacy workers’ knowledge of antihypertensive and anticonvulsant drugs for the prevention and management of PE/E is limited in Bangladesh. Urgent intervention for improved private pharmacy contributions to the public health is needed to mitigate any harmful practices or adverse outcomes from unauthorized, incorrectly prescribed, or misused drugs. As the majority of surveyed pharmacies and drug stores in this study are private and staffed with untrained workers with limited knowledge, it is imperative to implement comprehensive regulation and provide additional support and training to these pharmacy workers, who are often the first points of contact for health care for women and their families. Mandatory skills training should include awareness of potential adverse outcomes of drugs dispensed, and referrals for women with suspected hypertension or pregnancy-induced hypertension to a trained medical provider. Unauthorized pharmacy and drug staff should be made aware of the vital importance of reserving drug prescription solely to authorized, trained medical staff. To be effective, current law should be implemented properly, including strict licensing, routine and random checks by PCB, with fines as well as incentives to encourage private drug stores’ compliance. These efforts should be complemented by public education and demand creation campaigns.

## Supplementary information

**Additional file 1.**

## Data Availability

The data are not publicly available, we have assured the interviewees confidentiality and anonymity. However, data will be available only on request, under special circumstances after clearing with the ethics committee or authors group.

## Abbreviations

OR: Odds Ratio
AOR: Adjusted Odds Ratio
CI: Confidence Interval
PE: Pre-eclampsia
SPE: Severe Preeclampsia
E: Eclampsia
MgSO_4_: Magnesium Sulphate
UH&FWC: Union Health and Family Welfare Center
UHC: Upazila Health Complex
MCWC: Mother and Child Welfare Center
DH: District Hospital
FWV: Family Welfare Visitor
PHC: Primary Health Care
SACMO: Sub-assistant Community Medical Officer
WHO: World Health Organization
NIPORT: National Institute of Population Research and Training
BDHS: Bangladesh Demographic and Health Survey
PCB: Pharmacy Council of Bangladesh
IHT: Institute of Health Technology
MoHFW: Ministry of Health and Family Welfare
IRB: Institutional Review Board

## References

[1] Duley L (2009). The global impact of pre-eclampsia and eclampsia. Semin Perinatol.

[2] WHO. World Health Statistics. http://apps.who.int/iris/bitstream/10665/112738/1/9789240692671_eng.pdf?ua=1 (2014). Accessed 23 Aug 2016.

[3] WHO, UNICEF, UNFPA WBG, Division and the UNP. Trends in maternal mortality 1990 to 2015. http://apps.who.int/iris/bitstream/10665/194254/1/9789241565141_eng.pdf?ua=1 (2015). Accessed 3 May 2016.

[4] Say L, Chou D, Gemmill A (2014). Global causes of maternal death: a WHO systematic analysis. Lancet Glob Health.

[5] Kalim N, Anwar I, Khan J (2009). Postpartum Haemorrhage and eclampsia: differences in knowledge and care-seeking behaviour inTwo districts of Bangladesh. J Health Popul Nutr.

[6] National Institute of Population Research and Training (NIPORT), International Centre for Diarrhoeal Disease Research, Bangladesh (icddr,b), and MEASURE Evaluation (2017). *Bangladesh Maternal Mortality and Health Care Survey* 2016*:* preliminary report.

[7] Magee LA, Pels A, Helewa M, Rey E, Dadelszen PV (2014). Diagnosis, evaluation, and management of the hypertensive disorders of pregnancy, diagnosis, evaluation, and management of the hypertensive disorders of pregnancy. Pregnancy Hypertens.

[8] National Institute of Population Research and Training (NIPORT), Mitra and Associates, and ICF International (2013). Bangladesh demographic and health survey 2011, Dhaka, Bangladesh, and Rockville, Maryland, USA.

[9] Sarker SA, Nazma S, Most LB, Lobaba SL, Md FA, Md KH (2017). Pregnancy induced hypertension and associated factors among pregnant women. J Gynecol Women’s Health.

[10] Ahmed SM, Hossain MA (2007). Knowledge and practice of unqualified and semi-qualified allopathic providers in rural Bangladesh: implications for the HRH problem. Health Policy.

[11] Ahmed SM, Hossain MA, Chowdhury MR (2009). Informal sector providers in Bangladesh: how equipped are they to provide rational health care?. Health Policy Plan.

[12] Ahmed SM, Hossain MA, Chowdhury MR, Bhuiya AU (2011). The health workforce crisis in Bangladesh: shortage, inappropriate skill-mix, and inequitable distribution. Hum Resour Health.

[13] Ahmed SM, Islam QS (2012). Availability and rational use of drugs in primary healthcare facilities following the national drug policy of 1982: is Bangladesh on right track?. J Health Popul Nutr.

[14] Directorate General of Drug Administration: Allopathic retail pharmacy. http://dgda.gov.bd/index.php/pharmacies/allopathic-retail-pharmacy-view (2019). Accessed 28 May 2019.

[15] Ahmed SM, Naher N, Hossain T, Rawal LB (2017). Exploring the status of retail private drug shops in Bangladesh and action points for developing an accredited drug shop model: a facility based cross-sectional study. J Pharm Policy Pract.

[16] Lucas PJ, Uddin MR, Khisa N, Akther SMS, Unicomb L, Nahar P (2019). Pathways to antibiotics in Bangladesh: a qualitative study investigating how and when households access medicine including antibiotics for human or animals when they are ill. PLoS One.

[17] Huda FA, Afrin S, Sarker BK, Mahmood HR, Alam A (2017). Introduction and approval of manustrual regulation with medication in Bangladesh: a stakeholder analysis. STEPUP research report: Dhaka, icddr, b.

[18] Alam GM, Shahjamal MM, Al-Amin AQ, Azam MN (2013). State of pharmacy education in Bangladesh. Trop J Pharm Res.

[19] Hossain S, Roy S, Sultana K, Warren C (2019). Assessing the effect of primary health care level intervention in improving knowledge and practice on PE/E of PHC providers in Bangladesh.

[20] SIAPS (2015). Baseline study of private drug shops in Bangladesh: findings and recommendations. Submitted to the US Agency for international development by the Systems for Improved Access to pharmaceuticals and services (SIAPS) program.

[21] Saha S, Hossain MT (2017). Evaluation of medicines dispensing pattern of private pharmacies in Rajshahi, Bangladesh. BMC Health Serv Res.

[22] Pharmacy Council of Bangladesh (PCB). The Pharmacy Ordinance. http://bdlaws.minlaw.gov.bd/print_sections_all.php?id=513 (1976). Accessed 17 Feb 2019.

[23] Pharmacy Council of Bangladesh (PCB). Pharmacist’s code of ethics. Accessed 10 Apr 2019.

[24] James PR, Nelson-Piercy C (2004). Management of hypertension before, during, and after pregnancy. Heart..

[25] Duley L, Meher S, Jones L. Drugs for treatment of very high blood pressure during pregnancy. Cochrane Database Syst Rev. 2013;7:CD001449. 10.1002/14651858.CD001449.pub3 PMC7073408 23900968

[26] World Health Organization (2011). WHO recommendations for prevention and treatment of pre-eclampsia and eclampsia.

[27] Parr J, Lindeboom W, Khanam M, Sanders J, Koehlmoos TP (2012). Informal allopathic provider knowledge and practice regarding hypertension in urban and rural Bangladesh. PLoS One.

[28] Hadi MA, Karami NA, Al-Muwalid AS, Al-Otabi A, Al-Subahi E, Bamomen A (2016). Community pharmacists’knowledge, attitude, and practices towards dispensing antibiotics without prescription (dawp): a cross-sectional survey in Makkah province, Saudi Arabia. Int J Infect Dis.

[29] Roque F, Soares S, Breitenfeld L, Figueiras A, Herdeiro MT (2015). Influence of community pharmacists’ attitudes on antibiotic dispensing behavior: a cross-sectional study in Portugal. Clin Ther.

[30] Roque F, Soares S, Breitenfeld L, Gonzalez-Gonzalez C, Figueiras A, Herdeiro MT (2014). Portuguese community pharmacists’ attitudes to and knowledge of antibiotic misuse: questionnaire development and reliability. PLoS One.

[31] Reiss K, Footman K, Akora V, Liambila W, Ngo TD (2016). Pharmacy workers’ knowledge and provision of medication for termination of pregnancy in Kenya. J Fam Plann Reprod Health Care.

[32] Zawahir S, Lakmali N, Dhakshila N. Pharmacy practice in Sri Lanka. In: Ahmed F, Ibrahim M, Wertheimer A, editors. Pharmacy practice in developing countries: achievements and challenges. 1. 1 ed: Elsevier; 2016. p. 79–94.

[33] Kamuhabwa A, Jalal R (2011). Drug use in pregnancy: knowledge of drug dispensers and pregnant women in Dar Es Salaam, Tanzania. Indian J Pharm.

[34] Ross S, Maxwell S (2012). Prescribing and the core curriculum for tomorrow’s doctors: BPS curriculum in clinical pharmacology and prescribing for medical students. J Clin Pharmacol.

[35] Burkey BW, Holmes AP (2013). Evaluating medication use in pregnancy and lactation: what every pharmacist should know. J Pediatr Pharmacol Ther.

[36] Abalos E, Duley L, Steyn DW. Antihypertensive drug therapy for mild to moderate hypertension during pregnancy. Cochrane Database Syst Rev. 2014;(2):CD002252. 10.1002/14651858.CD002252.pub3 24504933

[37] WHO (2017). Managing complications in pregnancy and childbirth: a guide for midwives and doctors.

[38] Bains S, Kitutu FE, Rahhal A, Samaha RA, Wilby KJ, Rowe H (2014). Comparison of pharmacist knowledge, perceptions and training opportunities regarding maternal fetal medicine in Canada, Qatar and Uganda. Can Pharm J (Ott).

[39] Ahmed SM, Naher N, Hossain T, Rawal LB (2017). Exploring the status of retail private drug shops in Bangladesh and action points for developing an accredited drug shop model: a facility based cross-sectional study. J Pharm Policy Pract.

[40] Guillaume L, Cooper R, Avery A, Mitchell S, et al. Supplementary prescribing by community and primary care pharmacists: an analysis of PACT data, 2004–2006. J Clin Pharm Ther. 2008;33:11–6. 10.1111/j.1365-2710.2008.00869.x 18211611

[41] Huda FA, Mahmood HR, Alam A, Ahmmed F, Karim F, Sarker BK (2018). Provision of menstrual regulation with medication among pharmacies in three municipal districts of Bangladesh: a situation analysis. Contra..

[42] Sudhinaraset M, Ingram M, Lofthouse HK, Montagu D (2013). What is the role of informal healthcare providers in developing countries? A systematic review. PLoS One.

[43] Ahmed SM, Hossain MA, Chowdhury MR (2009). Informal sector providers in Bangladesh: how equipped are they to provide rational health care?. Health Policy Plan.

[44] Goundrey-Smith S (2018). The connected community pharmacy: benefits for healthcare and implications for health policy. Front Pharmacol.

[45] Vennue Foundation. Building a stronger global pharmacy workforce. http://www.vennue.org/our-model. Accessed 29 Apr 2019.

[46] International Pharmaceutical Federation (FIP). Pharmacy workforce intelligence: global trends reports. https://www.fip.org/www/streamfile.php?filename=fip/PharmacyEducation/Workforce_Report_2018.pdf. Accessed 12 Mar 2019.

[47] Asian Development Bank and the International Labour Organization. Women at work. https://www.adb.org/sites/default/files/publication/203906/women-work.pdf. Accessed 10 Apr 2019.

[48] Syed U, Khadka KA, Wall S (2008). Care-seeking practices in South Asia: using formative research to design program interventions to save newborn lives. J Perinatol.

[49] Kabir A, Huq MN, Al-Amin AQ, Alam GM (2012). Community participation on health and family planning programs in Bangladesh: the role of education and knowledge on HFP for plummeting pharmaceutical costing. Int J Pharm.

[50] Karim R, Lindberg L, Wamala S, Emmelin M (2018). Men’s perceptions of Women’s participation in development initiatives in rural Bangladesh. Am J Men’s Health.

[51] International Pharmaceutical Federation (FIP). Pharmacy at a glance 2015–2017. https://www.fip.org/file/1348. Accessed 13 Mar 2019.

