# Supplementary material for: Knowledge of pharmacy workers on antihypertensive and anticonvulsant drugs for managing pre-eclampsia and eclampsia in Bangladesh
Source: BMC Health Serv Res. 2020 Sep 7;20:838. doi: 10.1186/s12913-020-05685-6 (PMC7487640; doi:10.1186/s12913-020-05685-6)
Supplement: Supplementary file 1 — Additional file 1. [file 12913_2020_5685_MOESM1_ESM.pdf]

Questionnaire number [M_ | _| _ | _ | _ |_]

MEDICAL STORES AND PHARMACIES

FACILITY IDENTIFICATION		
Country: Bangladesh		
District: _____________	Upazila: ______________________	Union: ______________________
Facility name: ____		
Facility type/drug stores	1= Tertiary Hospital/Medical college<br>2= District Hospital/Sadar Hospital<br>3= Maternal & Child Welfare Centre (MCWC)<br>4= Upazila Health Complex (UHC)<br>5= Union Health & Family Welfare Centre (UH&FWC)<br>6. Private pharmacy/drug store<br>7= Others (*specify*)______________________	
Type of sector	1= Government of Bangladesh (GOB) pharmacy/drug store<br>2= Private pharmacy/drug store<br>7= Others (*specify*)________________________	

INTERVIEW OUTCOMES			
**INTERVIEW DATE**(DAY / MONTH / YEAR) e.g. 06/03/16		[______/______/______]	
**INTERVIEW RESULT**	1= Completed<br>2= Partially Completed<br>3= Refused<br>7= Others (*specify*)________________________	[___|___]	
**INTERVIEWER'S NAME**		Interviewers code	

	**SUPERVISOR**	**EDITED BY**	**ENTERED BY**
Name:	______________________	______________________	______________________
Date:	______________________	______________________	______________________

Time Interview Started: [___|___:___|___] **[RECORD TIME IN 24-HOUR CLOCK]**

TIME INTERVIEW ENDED: [___|___:___|___] **[RECORD TIME IN 24-HOUR CLOCK**

Section 1: General Information
(INS: Please circle or write)

Q101	Respondent sex	1. Male<br>2. Female
Q102	Respondent age in completed years	Write in: __________________
Q103	What is your position at this facility/drug store?<br>*(Alternatively: What is your title?)*	01. Pharmacist<br>02. FWV<br>03. SACMO<br>04. Supply Officer<br>05. Store Keeper/Manager<br>06. Drug seller<br>07. Drug store owner<br>77. Other *(specify)* ______________________
Q104	What is your basic medical training?<br>*(Do not read. Listen to the answers and check all that are applicable.)*	01. Pharmacist/diploma pharmacist<br>02. Certificate course<br>03 Learned from experience<br>04. Informal healthcare (village doctor/Pollichikitshok)<br>7. Other (specify) _____________________
Q105	How long have you been serving this position?	1. 0–6 months<br>2. 7–12 months<br>3. 1–5 years<br>4. >5 years
Q106	How long have you been employed at this facility/drug store?	1. 0–6 months<br>2. 7–12 months<br>3. 1–5 years<br>4. >5 years

Section 2: Knowledge on Antihypertensive Drug

Q201	Are you aware with the medicines used to treat hypertension?	1. Yes<br>2. No (Skip to Q203)
Q202	If yes, what are the medicines used to treat or prescribe for hypertension?	01. Propanol<br>02. Atenolol<br>03. Alpha Methyldopa<br>04. Hydralazine<br>05. Nifedipine<br>06. Diazepam<br>07. Losartan Potassium<br>77. Other *(specify)* _______________________
Q203	Do you know what antihypertensive drugs are safe to use during pregnancy period?	1. Yes<br>2. No (Skip to Q205)

Q204	If yes, what are the name of those anti-hypertensive drugs?	1. Alpha Methyldopa<br>2. Hydralazine<br>3. Nifedipine<br>4. Diazepam<br>5. Labetalol<br>7. Other *(specify)* ________________________	
Q205	Are you familiar with the medicines used for treatment or prescribe of pre-eclampsia?	1. Yes<br>2. No (Skip to Q207)	
Q206	If yes, what are those medicines?	1. Alpha Methyldopa<br>2. Hydralazine<br>3. Nifedipine<br>4. Diazepam<br>5. Labetalol<br>6. Magnesium sulphate<br>7. Other *(specify)* ________________________	
Q207	Are you familiar with the medicines for treatment or prescribe of seizures/convulsion during pregnancy?	1. Yes<br>2. No (Skip to Q209)	
Q208	If yes, what are those medicines?	1. Magnesium sulphate<br>2. Diazepam<br>3. Phenobarbitone<br>4. Phenytoin<br>7. Others (specify)____________________	
Q209	Have you heard about a drug called calcium gluconate?	1. Yes<br>2. No (Skip to Q211)	
Q210	If yes, in which situation it is used?	1. To manage toxicity of magnesium sulphate<br>7. Others (specify):________________________<br>9. Do not know	
Q211	What are the storage guidelines for antihypertensive drugs?	1. Room temperature<br>2. Dry place<br>7. Other (*specify)* ____________________<br>9. Do not know	
Q212	What are the storage guidelines for magnesium sulfate, and calcium gluconate?	**Magnesium Sulfate**<br>1. Keep in a box protected from light and freezing temperatures<br>2. Keep in a box protected from light and room temperatures<br>7. Other (specify):_____________<br>9. Do not know	**Calcium Gluconate**<br>1. Keep in a box protected from light and freezing temperatures<br>2. Keep in a box protected from light and room temperatures<br>7. Other (specify):_____________<br>9. Do not know

Q213	What is the shelf life of magnesium sulphate?	1. 6 months<br>2. 1 year<br>3. 2 years<br>7. Other (specify): ______________________
Q214	What are the items to be checked upon receipt of a medicine?<br>*(Do not lead the interview. Circle all responses mentioned spontaneously.)*	1. Form<br>2. Expiration date<br>3. Administration route<br>3. Quality<br>7. Other (specify):______________________<br>9. Do not know

Section 3: Procurement/orders

Q301	Do you personally manage the stock of anti-hypertensive, severe pre-eclampsia and eclampsia treatment medicines in your facility/drug store?	1. Yes<br>2. No<br><br>If no write in title of person who does this<br>___________________________________
Q302	How do you put orders for antihypertensive drug when you need it?	1. Give estimate to the facility manager in written<br>2. Give estimate to the facility manager in verbal<br>3. Place requirement in the procurement committee<br>4. It comes regularly in an interval from central level<br>5. Purchase locally<br>7. Others (*specify*):_______________________<br>9. Do not know
Q303	How do you put orders for magnesium sulphate and calcium gluconate when you need it?	01. Give estimate to the facility manager in written<br>02. Give estimate to the facility manager in verbal<br>03. Place requirement in the procurement committee<br>04. It comes regularly in an interval from central level<br>05. Purchase locally<br>06. Do not keep this drug<br>77. Others (*specify*):_______________________<br>99. Do not know
Q304	How do you estimate the facility's need of anti-hypertensives, severe pre-eclampsia and eclampsia treatment medicines requirements?<br><br>*(Do not lead the interview. Circle all responses mentioned spontaneously. The respondent must mention each aspect of the responses given.)*	01. General experiences<br>02. Doctors advise<br>03. Based on 1/3/6/12 months consumption<br>04. Stock on hand<br>06. It comes regularly in an interval from central level<br>07. Purchase in a lump sum amount<br>77. Other *(specify)*_______________________<br>99. Do not know

Q305	What is the frequency of orders/purchase for anti-hypertensive drugs?<br><br>*(Do not lead the interview. Check all responses mentioned spontaneously. The respondent must mention each aspect of the responses given.)*	01. Every week<br>02. Twice a month<br>03. Once a month<br>04. Once in 3 months<br>05. Once in 6 months<br>06. Once a year<br>07. When required/stock exhausted<br>77. Other *(specify)* ___________________________<br>99. Do not know	
Q306	What is the frequency of orders for magnesium sulphate and calcium gluconate?<br>*(Do not lead the interview. Check all responses mentioned spontaneously. The respondent must mention each aspect of the responses given.)*	**Magnesium Sulphate**<br>01. Every week<br>02. Twice a month<br>03. Once a month<br>04. Once in 3 months<br>05. Once in 6 months<br>06. Once a year<br>07. When necessary<br>08. Do not keep this drug<br>77. Other *(specify)*:_______<br>99. Do not know	**Calcium Gluconate**<br>01. Every week<br>02. Twice a month<br>03. Once a month<br>04. Once in 3 months<br>05. Once in 6 months<br>06. Once a year<br>07. When necessary<br>08. Do not keep this drug<br>77. Other *(specify)*:_______<br>99. Do not know
Q307<br>**<u>GOB</u>**	Are the orders sent/filled in requested amount?	1. Yes<br>2. No<br>8. Not applicable	
Q308<br>**<u>GOB</u>**	If no, why not?	1. Central-level stock-outs<br>2. Lack of budget at central level<br>3. Order not issued on time<br>4. Not applicable<br>7. Other *(specify)*:________________________<br>9. Do not know	
Q309<br>**<u>GOB</u>**	Are the orders sent/filled in requested time?	1. Yes<br>2. No<br>8. Not applicable	
Q310<br>**<u>GOB</u>**	If no, why not?	1. Central-level stock-outs<br>2. Lack of budget at central level<br>3. Order not issued on time<br>4. Not applicable<br>7. Other *(specify)*:________________________<br>9. Do not know	
Q311	Where do you obtain your supply of anti-hypertensive drug, calcium carbonate, calcium lactate, magnesium sulphate and calcium gluconate?	01. Government Central Medical Store of Drug (CMSD)<br>02. Regional Warehouse<br>03. District Warehouse<br>04. Private wholesalers<br>05. Private Pharmacies<br>06. Medical Store<br>07. Donations<br>08. NGO<br>77. Other *(specify)*:_________________________	

Section 4: Current Status of Stock, Practice and Management of Stock

<table>
<tr><td>Q401
<u>GOB</u></td><td>Do you have any drug store/room in your facility?</td><td colspan="4">1. Yes
2. No</td></tr>
<tr><td>Q402</td><td>Do you have any refrigerator for storage of drugs?</td><td colspan="4">1. Yes
2. No</td></tr>
<tr><td>Q403</td><td>Please, check the condition of the room:</td><td colspan="4">1. Spacious
2. Congested
3. Well ventilated
4. Damped
5. Light available
6. Dark/light not available
7. Medicines are well managed and orderly kept
8. Medicines are haphazardly kept
77. Others (specify):_____________
Comments (if any):_______________</td></tr>
<tr><td>Q404</td><td>What are the anti-hypertensive medicines are currently available in your store/pharmacy?</td><td colspan="4">01. Atenolol
02. Propranolol
03. Aspirin
04. Alpha Methyldopa
05. Hydralazine
06. Nifedipine
07. Labetalol
08. Diazepam
09. Losartan Potassium
10. Thyazides
77. Other (specify): ____________</td></tr>
<tr><td rowspan="7">Q405</td><td rowspan="7">Is following anti-hypertensives are available in this facility/drug store?</td><td>Medicine</td><td>Yes</td><td>No</td><td>Do not know</td></tr>
<tr><td>1. Alpha Methyldopa</td><td>1</td><td>2</td><td>99</td></tr>
<tr><td>2. Hydralazine</td><td>1</td><td>2</td><td>99</td></tr>
<tr><td>3. Nifedipine</td><td>1</td><td>2</td><td>99</td></tr>
<tr><td>4. Labetelol</td><td>1</td><td>2</td><td>99</td></tr>
<tr><td>5. Calcium Carbonate</td><td>1</td><td>2</td><td>99</td></tr>
<tr><td>6. Calcium Lactate</td><td>1</td><td>2</td><td>99</td></tr>
<tr><td>Q406</td><td>Do you have stock of magnesium sulphate and calcium gluconate?</td><td colspan="3">Magnesium Sulphate
1. Yes
2. No</td><td>Calcium Gluconate
1. Yes
2. No</td></tr>
<tr><td>Q407</td><td>When the last time you received or purchase anti-hypertensive medicines?
(any one of the antihypertensive)</td><td colspan="4">01. Every week
02.Every month
03.3months back
04. 6 months back
05.1year back
06.2 years back
07.Never</td></tr>
</table>

<table>
<tr><td></td><td colspan="2"></td><td colspan="3">08.Not applicable
77. Others (specify):______
Put exact date from register:
________________________</td></tr>
<tr><td rowspan="7">Q408</td><td colspan="2" rowspan="7">When the last time you received or purchase the following anti-hypertensive medicine?</td><td>Medicine</td><td>Date</td><td>Do not know</td></tr>
<tr><td>1. Alpha Methyldopa</td><td>---/----/---/</td><td>99</td></tr>
<tr><td>2. Hydralazine</td><td>---/----/---/</td><td>99</td></tr>
<tr><td>3. Nifedipine</td><td>---/----/---/</td><td>99</td></tr>
<tr><td>4. Labetelol</td><td>---/----/---/</td><td>99</td></tr>
<tr><td>5. Calcium Carbonate</td><td>---/----/---/</td><td>99</td></tr>
<tr><td>6. Calcium Lactate</td><td>---/----/---/</td><td>99</td></tr>
<tr><td>Q409</td><td>When the last time you received or purchase magnesium sulphate and calcium gluconate?</td><td>**Magnesium sulphate**
1. Every week
2. Every month
3. 3 months back
4. 6 months back
5. 1 year back
6. 2 years back
7. Never
8. Not applicable
77. Others (specify):______
Put exact date from register:
________________________</td><td colspan="3">**Calcium gluconate**
1. Every week
2. Every month
3. 3 months back
4. 6 months back
5. 1 year back
6. 2 years back
7. Never
8. Not applicable
77. Others (specify):______
Put exact date from register:
________________________</td></tr>
<tr><td>Q410
GOB</td><td colspan="2">Do you have stock registers for all the medicines and supplies that are managed at this facility/drug store?</td><td colspan="3">1. Yes
2. No (*Please note the stock management tools available)*
________________________________</td></tr>
<tr><td>Q411
GOB</td><td colspan="2">If yes, are these registers up-to-date?

(Observe these materials before answering the question.)</td><td colspan="3">1. Yes
2. No
7. Others (*specify*):____________</td></tr>
<tr><td>Q412
GOB</td><td colspan="2">How frequently are stock registers updated?
(skip if no stock registers at facility)</td><td colspan="3">1. Daily
2. Weekly
3. Monthly
4. Bi-monthly (every two weeks)
5. Not applicable
7. Other *(specify)*: ____________</td></tr>
<tr><td>Q413
GOB</td><td colspan="2">Is there a system for recording the transfer of anti-hypertensives between the pharmacy and the delivery room?</td><td colspan="3">1. Yes
2. No
8. Do not know</td></tr>
</table>

Section 5: Submission of Reports

Q501<br>**GOB**	Do you prepare reports on the consumption and inventory positions of medicines?	1. Yes<br>2. No (Skip to Section 6)
Q502<br>**GOB**	A. Where do you send the reports? ____<br><br>B. How do you send them? ____<br><br>C. What is the frequency? Daily /___/ Weekly /___/ Monthly /___/<br>Quarterly /___/ Biannual /___/ Annual /___/ Other (to be specified) /___/<br><br>D. What information do the reports contain?<br>a. Quantity received /___/<br>b. Quantity distributed /___/<br>c. Quantity expired /___/<br>d. Quantity in stock /___/<br>7. Other (to be specified) ________________________________	

Section 6: Supervision

Q601<br>**GOB**	Do you have a direct supervisor?	1. Yes<br>2. No (skip to Q603)
Q602<br>**GOB**	Who is your direct supervisor?	01. Hospital administrator/manager/in-charge<br>02. Civil surgeon<br>03. DD-FP<br>04. UH&FPO<br>05. UFPO<br>06. SACMO<br>07. FWV<br>08. MO-MCH-FP/Medical Officer<br>09. Regional pharmacist/store keeper<br>10. District pharmacist/store keeper<br>11. Central Store keeper<br>12. Do not have any supervisor<br>77. Other (specify):______________________<br>99. Do not know
Q603<br>**GOB**	When was the last time your direct supervisor visited you?	1. This month<br>2. Last month<br>3. Three months ago<br>4. Six months ago<br>5. Never (Skip to Q 701)<br>7. Other *(specify)* ______________<br>9. Do not know/not sure

Q604<br>**GOB**	What did your supervisor see/review when he/she was here?	1. Check stock register<br>2. Look at consumption trends<br>3. Look at storage conditions<br>4. Consolidate requirements<br>5. Check physical stock<br>6. Observe the management of expired and damaged products<br>7. Other *(specify)*:_________________________
Q605<br>**GOB**	Did your supervisor shared his/her comments and reactions with you?	1. Yes<br>2. No

Section 7: Training

Q701	Have you been trained on the management of medicines in the last 12 months?	1. Yes<br>2. No
Q702	Have you ever been trained on the management of medicines?	1. Yes<br>2. No (Skip Q704)
Q703	If yes, have you had the opportunity to put into practice what you learned?	1. Yes<br>2. No
Q704	Do you think you need practical training to feel more confident using standardized procedures for the management of medicines?	1. Yes<br>2. No (Skip Q801)
Q705	If yes, on what aspects of the stock management do you need training?	1. Storage<br>2. Preservation<br>3. Transport<br>4. Use<br>5. All subjects<br>7. Others (*specify*) __________________

Section 8: Tools

Q801	Do you have a copy of the National Essential Drug List?<br>*(Ask to see the NEDL)*	1. Yes (*Note year of the NEDL*<br>______________________________<br><br>2. No<br>9. Do not know
Q802	Does your facility have worksheets, such as job aids, on the management of medicines?	1. Yes<br>2. No (if no end the interview)<br>9. Do not know
Q803	If yes, where are these worksheets / job aids? ______________________________________<br>Please specify which ones are available ____	
Q804	If yes, are these sheets / job aids easy to understand?<br>Yes/___/ No/___ /	1. Yes<br>2. No
Q805	If no, what are the problems in understanding these sheets/job aids?<br>____	
Q806	During supervision, do your supervisors assess your comprehension of these worksheets / job aids?	1. Yes<br>2. No

Interviewer comments:
